# Incorporating long-range dependence and fractal features in turbulence spectra

**DOI:** 10.1038/s41598-025-16950-1

**Published:** 2025-08-27

**Authors:** Shyuan Cheng, Yaswanth Sai Jetti, Vincent S. Neary, Martin Ostoja-Starzewski, Leonardo P. Chamorro

**Affiliations:** 1 Department of Mechanical Science and Engineering, University of Illinois, Urbana, IL USA; 2 Sandia National Laboratories, Albuquerque, New Mexico USA; 3 Beckman Institute, University of Illinois, Urbana, IL USA; 4 Department of Aerospace Engineering, University of Illinois, Urbana, IL USA; 5 Department of Civil and Environmental Engineering, University of Illinois, Urbana, IL USA; 6 Department of Earth Science and Environmental Change, University of Illinois, Urbana, IL USA

**Keywords:** Civil engineering, Mechanical engineering

## Abstract

We introduce an advanced turbulence spectrum model developed from mathematical foundations from a covariance function class and empirically validated using extensive field data. This model captures the complex dynamics of long-range dependence, and fractal characteristics prevalent in riverine and atmospheric boundary layer (ABL) flows that are ignored by classical spectrum models, such as IEC (International Electrotechnical Commission) von Kármán and Kaimal model. The model delineates scaling behaviors across distinct frequency bands and offers substantial flexibility through five well-defined parameters each characterizing a distinct physical aspect of the velocity time series. A detailed procedure for obtaining each parameter from time series data is outlined. The comprehensive validations with field data from tidal currents and ABL flows substantiate the model’s fidelity in accurately replicating observed phenomena. This validation establishes the reliability of the proposed model and, when incorporated into stochastic full-field simulators such as TurbSim, demonstrates its potential to advance the predictive modeling and analysis of turbulent flows in environmental science and engineering contexts.

## Introduction

The turbulence spectrum serves as a pivotal tool to describe the kinetic energy distribution across the scales of flow fluctuations. Its accurate representation is essential in numerous engineering fields and environmental disciplines. Velocity spectral models are central to estimating unsteady structural loading, modeling the transport of particles and scalars, and generating flow fields in high-fidelity simulations.

Among the various empirical models formulated based on wind data, those by Davenport^1^, Harris^2^, Hino^3^, Kaimal^4^, and von Kármán^5^ are commonly used. These models, particularly the von Kármán and Kaimal models, simulate the velocity spectrum in the inertial subrange following Kolmogorov scaling. Such models underpin advanced stochastic unsteady turbulent inflow simulators like TurbSim, developed by the National Renewable Energy Laboratory (NREL)^6,7^, for critical applications including turbine load analysis, imbalance fault diagnosis, and Levelized Cost of Energy (LCOE) estimation^7–11^. However, these formulations may fail to capture significant aspects of turbulence, such as long-range dependence (LRD) effects, fractal behavior, and intermittent patterns. Long-range dependence (LRD) characterizes the persistence of a flow, specifically, how future states depend on past events due to memory effects, and is quantified by the Hurst exponent *H*. Standard spectral models typically assume that turbulent flows have no memory, implying statistical independence between past and future velocity increments. This assumption inherently leads to a flat scaling in the energy-containing range^12–14^. In contrast, most environmental flows exhibit LRD, driven by factors such as climatic variability^15–17^, large-scale weather systems^18^, external forcing mechanisms^19^, and atmospheric stability^20^. These effects contribute to a negative spectral slope in the energy-containing range, which classical turbulence models fail to capture accurately^12–14,21^. The fractal dimension *D* reflects the self-affinity and geometric complexity of the flow, and environmental turbulence often exhibits multifractality, with *D* varying across scales. These multifractal features are closely tied to turbulence intermittency and stability conditions^22–24^. The omission of LRD and fractal behaviors greatly influences the scaling within energy-containing regions. This limitation can lead to underestimating the spectral energy content, particularly in the low-frequency range, a critical gap that needs to be addressed in detail^25–27^.

Studying the turbulence spectrum and associated formulations has long been a major target in science and engineering. Foundational work by Kolmogorov^28,29^ provided the basis for the energy spectrum of turbulence, offering unique insights into its energy distribution. However, characterizing the features of the turbulence spectrum in distinct frequency regions remains a significant challenge, demanding innovative approaches to capture contributing phenomena effectively. Recent studies have also revealed new non-classical behaviors in the pre-inertial range of wall-bounded turbulence that further challenge the sufficiency of existing spectral models. For example, Ali and Dey^30^ identified a zeroth-law scaling in the helicity spectrum, $H(k) \sim k^0$, arising from the combined effects of energy and helicity transfer induced by wall-attached superstructures.

Recent progress in spectral formulations of random fields has shown promising results in overcoming these limitations (Lim *et al.*^31^; Faouzi *et al.*^32^). These new models provide practical tools to extract and interpret velocity field data effectively while incorporating fractal dimensions (*D*) and Hurst parameters (*H*) have introduced novel ways to analyze the intermittent and multi-scale turbulence processes^33^. We introduce a velocity spectral model derived from the parametric family of fractal and Hurst effect decouplers formulated by Jetti *et al.*^34^.

This formulation accounts for short- and long-range dependencies, as determined by its parametric settings. This new class of velocity spectrum model requires similar input parameters to those used in traditional turbulence spectrum models, e.g., the IEC von Kármán isotropic model, and IEC Kaimal model^35^. These parameters can be easily obtained from measured or simulated time series. We demonstrate that the new model returns superior scale-dependent energy distribution accuracy compared to those obtained from traditional empirical spectrum models. This approach can also provide the basis to develop generalized two-dimensional^36^ and three-dimensional^37^ counterparts that account for the aforementioned phenomena.

The manuscript is organized as follows. The new class of covariance functions and their spectral representation are introduced in Section 1, followed by the description of the procedure to obtain the required model parameters in Section 2; the detrending, multifractal analysis, and model performance evaluation using the atmospheric boundary layer (ABL) and tidal flow datasets are discussed in Section 3; and the main conclusions are provided in Section 4.

**Fig. 1:**
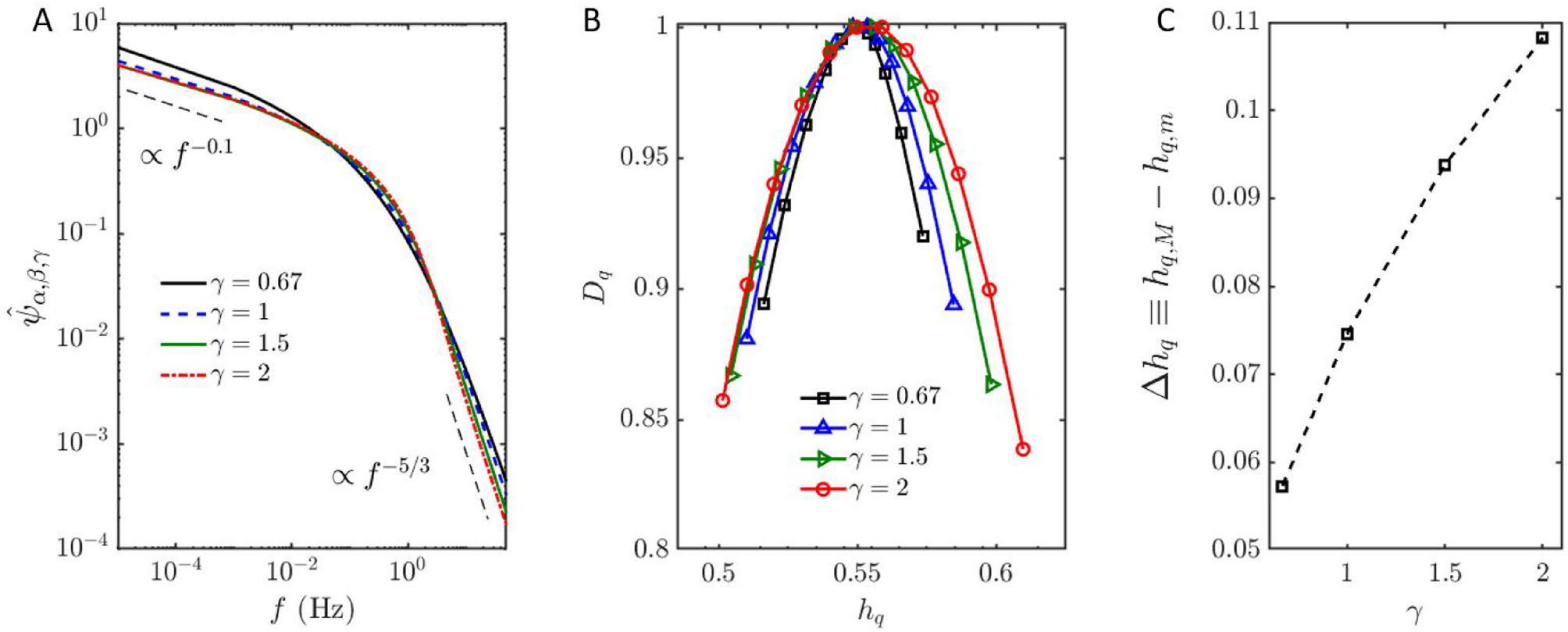
(**a**) Spectral distributions for long-range dependence covariance model sharing *H* ($\gamma \beta$ = 0.9) and *D* ($\gamma \alpha$ = 2/3) but different $\gamma$. (**b**) Multifractal spectrum showing $q-$th order singularity exponent ($h_q$) versus $q-$th order singularity dimension $D_q$ obtained from simulated time series with various $\gamma$, and (**c**) multifractal spectrum width ($\Delta h_q$) versus $\gamma$ for various $\gamma$ realizations; the simulated time series shared $d=1$, $\gamma \alpha =2/3$, and $\gamma \beta =0.9.$.

## Base formulation and contributing factors

Let us first consider the parametric family of isotropic functions proposed by Jetti *et al.*^34^, where each parameter must be rigorously linked to the physical processes they represent. The fundamental formulation is defined as follows:

1 $$\begin{aligned} \psi _{\alpha ,\beta ,\gamma }(x) = \sigma ^2 (1 - ( 1 + (x/c)^{-\gamma }) ^{-\alpha })^{\beta }, \qquad x \ge 0, \end{aligned}$$

where $\sigma ^2$ represents the covariance, and *c* is a scaling parameter. The function $\psi _{\alpha ,\beta ,\gamma }(\cdot )$ qualifies as an isotropic covariance function in $\mathbb {R}^d$ for any dimension $d = 1, 2, \ldots$, provided $\alpha \in (0,1]$, $\beta > 0$, and $\gamma \in (0,2]$. Importantly, $\psi _{\alpha ,\beta ,\gamma }(x) \ge 0$, ensuring that it models only positive covariances and does not represent negative correlations. Note that, in contrast, the covariance function of the classical Von Kármán model is complex, involving modified Bessel functions of the second kind; see Laudani *et al.*^33^. As discussed next, the quantity $\gamma \alpha$ is related to the fractal behavior or short-range fluctuations of the random-like field, here the turbulent velocity fluctuations. The quantity $\gamma \beta$ is directly linked to the Hurst exponent used as a measure of long-term memory of time series^16,17^. The parameter $\gamma$ controls the transition between the long-range and short-range scaling behaviors. The parameter *c* as noted is a scaling parameter and is related to the integral length scale (or the correlation length) of the underlying random-like field. For classical turbulence spectrum models (i.e., IEC von Kármán and Kaimal model) $\gamma \alpha$ is set to 5/3 for one-dimensional velocity spectra to enforce the Kolmogorov $-5/3$ slope in the inertial subrange and set $\gamma \beta = 0.5$ assuming that the past and future velocity fluctuations in the flows are independent; while $\gamma \beta = 1/3$ correspond to the Kolmogorov scaling law^38^ under self-affinity assumption.

Jetti *et al.*^34^ demonstrated that $\psi _{\alpha ,\beta ,\gamma } \in L_1(\mathbb {R}^d)$ when $\gamma \beta > d$, characterizing the function as exhibiting short-range dependence (SRD). Conversely, when $\gamma \beta \le d$, the function displays LRD, which is more commonly observed in turbulence field data and is the main focus of the manuscript. For further insights into SRD, refer to the supplementary information.

The associated spectral density of this parametric family is expressed as follows^39^:

2 $$\begin{aligned} \widehat{\psi }_{\alpha ,\beta ,\gamma }(f)&= -\sigma ^2\frac{ f^{1-\frac{d}{2}}}{2^{\frac{d}{2}-1}\pi ^{\frac{d}{2}+1}} \text {Im} \int _{0}^{\infty }K_{\frac{d}{2}-1}(ft)(1 - (1 + (i\frac{t}{c})^{-\gamma })^{-\alpha } )^{\beta }t^{d/2}\,\text {d}t,\ \end{aligned}$$

where $K_{\nu }$ denotes the modified Bessel function of the second kind of order $\nu$. For a valid covariance function $\psi _{\alpha ,\beta ,\gamma }$, we have

3 $$\begin{aligned} 1-\frac{\psi _{\alpha ,\beta ,\gamma }(x)}{\sigma ^2}&\sim \frac{\beta }{c^{\gamma \alpha }}x^{\gamma \alpha }, x\rightarrow 0 \end{aligned}$$

4 $$\begin{aligned} \frac{\psi _{\alpha ,\beta ,\gamma }(x)}{\sigma ^2}&\sim \alpha ^{\beta } c^{\gamma \beta } x^{-\gamma \beta }, x\rightarrow \infty , \end{aligned}$$

Here, $g \sim h$ indicates that the function *g* behaves like *h* under the specified asymptotic limit. For detailed asymptotic SRD results, refer to the Supplementary Material. The low and high-frequency limits of the spectral density $\widehat{\psi }_{\alpha ,\beta ,\gamma }$ for LRD are given by:

5 $$\begin{aligned} \widehat{\psi }_{\alpha ,\beta ,\gamma }(f)&\sim \sigma ^2C_0 (fc)^{\gamma \beta -d} for \gamma \beta \in \left( \frac{d-1}{2}, d\right) , f\rightarrow 0, \end{aligned}$$

6 $$\begin{aligned} \widehat{\psi }_{\alpha ,\beta ,\gamma }(f)&\sim \sigma ^2C_1(fc)^{-d-\gamma \alpha } for f\rightarrow \infty , \end{aligned}$$

where $C_0$ and $C_1$ are functions of $\alpha$, $\beta$, $\gamma$, *c*, and *d*. For completeness, the explicit expressions for $C_0$ and $C_1$ are given below:

7 $$\begin{aligned} C_0&= c\frac{2^{-\gamma \beta }\alpha ^{\beta }\Gamma \left( \frac{d}{2}-\frac{\gamma \beta }{2}\right) }{\pi ^{d/2}\Gamma \left( \frac{\gamma \beta }{2}\right) },\end{aligned}$$

8 $$\begin{aligned} C_1&= c\frac{2^{\gamma \alpha -1}\alpha \beta \gamma }{\pi ^{d/2}}\frac{\Gamma \left( \frac{d}{2}+\frac{\gamma \alpha }{2}\right) }{\Gamma \left( 1-\frac{\gamma \alpha }{2}\right) }. \end{aligned}$$

The asymptotic behavior of the covariance function reveals the fractal and Hurst properties inherent in the model, where the fractal dimension quantifies the degree of scale invariance within the time series, and the Hurst describes the long-range dependence effect, both of which are critical for characterizing turbulence data. From the derived asymptotic relations, the fractal dimension *D* and Hurst parameter *H* can be uniquely determined using the equations below:

9 $$\begin{aligned} D = d + 1 - \frac{\gamma \alpha }{2}, \end{aligned}$$

10 $$\begin{aligned} H = 1 - \frac{\gamma \beta }{2}. \end{aligned}$$

Equations 9 and 10 illustrate that $\alpha$ and $\beta$ facilitate tuning *D* and *H* (i.e., the high and low-frequency limits) given a specific $\gamma$. The influence of varying $\gamma$ is explored by generating four unity variance random time series, each with different $\gamma$ values while maintaining a constant $\gamma \alpha = 2/3$, $\gamma \beta = 0.9$, and $c=1$. The values of $\gamma$ are set to [2/3, 1, 3/2, 2]. These values are chosen to ensure the time series correspond to having $D=5/3$ for one-dimensional turbulent velocity time series^40–42^ and represent an LRD process. The time series were generated using the circulant embedding method (CEM)^43^, with each simulated time series being of total length $2^{16}$.

The spectra of these four simulated time series are depicted in Fig. 1a, demonstrating that increasing $\gamma$ sharpens the transition band between the *H* and *D* determined asymptotic high and low-frequency behavior. Figure 1b further elucidates the relationship between $\gamma$ and the degree of multifractality by looking into their multifractal spectra, showing that a higher $\gamma$ results in a sharper transition by increasing the multifractality^44,45^ (more information regarding multifractal spectrum is provided Section 2). An empirical correlation between $\gamma$ and the multifractal spectrum width ($\Delta h_q$) is presented in Fig. 1c. It illustrates a lower bound for the multifractal spectrum width at the highest $\gamma$ value ($\Delta h_q \approx 0.11$ at $\gamma = 2$), indicating that the current class of covariance functions only allow limited source of multifractility at the transition band (Equation 1). The potential inclusion of non-Gaussian distribution processes and the expansion to a full multifractal model that includes intermittency effect are considerations left for future work.

**Fig. 2:**
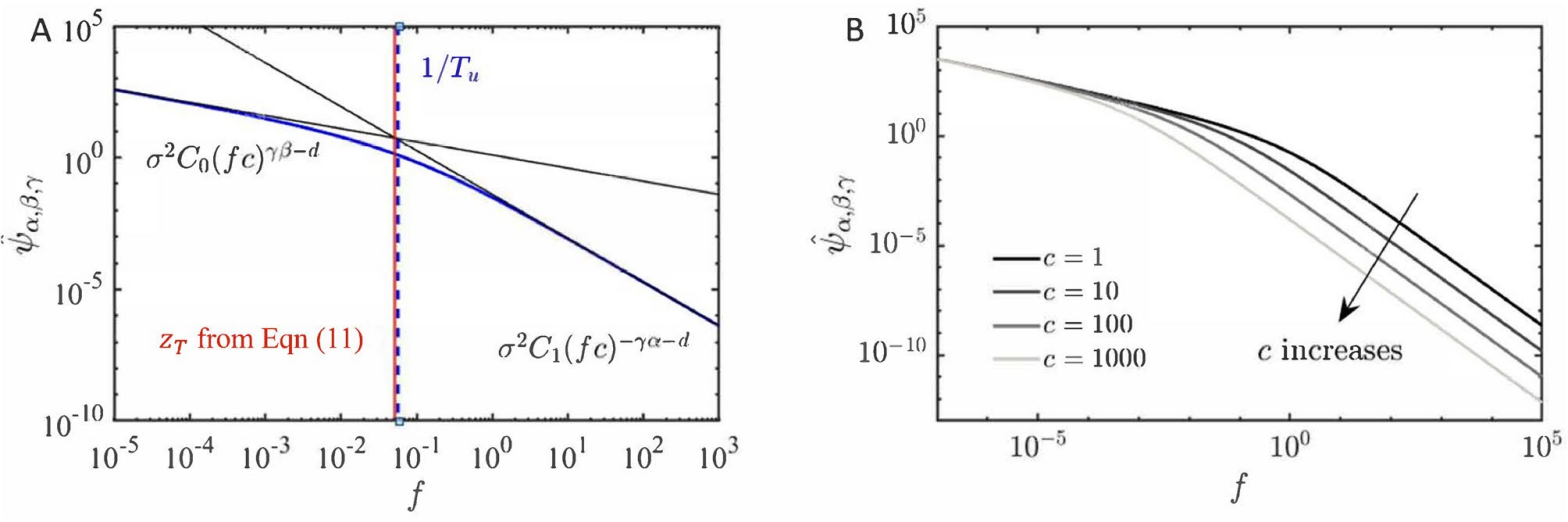
Spectral density $\widehat{\psi }_{\alpha ,\beta ,\gamma }(f)$ for a long-range dependent covariance model. (**a**) $\gamma \beta =0.5$, $c=10$, and $\sigma ^2=1$, illustrating the transition frequency $z_T \approx 1/T_u$. Black lines indicate the asymptotic low and high-frequency behaviors of $\widehat{\psi }_{\alpha ,\beta ,\gamma }(f)$. (**b**) $\gamma \beta =0.5$ with varying scaling constants *c*, such that $\sigma ^2C_0c^{\gamma \beta -1}=1$. In both cases, $d=1$, $\gamma \alpha =2/3$, and $\gamma =2/3$.

Tuning the scaling constant *c* allows control over the transition band frequency within the turbulence spectrum; it is related to the integral time-length scale ($T_u,L_u$) of the velocity time series. The integral time-length scale represents the transition point where the spectral density function shifts from low to high-frequency behavior. This transition point can be approximated as the intersection of the asymptotic relations in Equations 5 and 6 when the covariance function exhibits LRD. Thus, the transition frequency is given by:

11 $$\begin{aligned} z_T = \frac{1}{c}\left( \frac{C_1}{C_0}\right) ^{1/\gamma (\alpha +\beta )}. \end{aligned}$$

For example, consider the spectral density $\widehat{\psi }_{\alpha ,\beta ,\gamma }(f)$ when $d=1$, $\gamma \beta =0.5$, $\gamma \alpha =2/3$, $\gamma =2/3$, $\sigma ^2=1$, and $c=10$, as illustrated in Fig. 2a. The transition frequency $z_T$, calculated using Equation 11 and the transition band frequency approximated by the inverse of the integral time scale $T_u$, are also included, confirming that $z_T \approx 1/T_u$ (and $z_T \approx 1/L_u$ for the wavenumber spectrum). When $d=1$, the integral length scale, $L_u$, is obtained from the covariance function as:

12 $$\begin{aligned} L_u = \frac{1}{\sigma ^2}\int _{0}^{L_0} \psi _{\alpha ,\beta ,\gamma }(x) \,dx\, \end{aligned}$$

and can also be calculated from a velocity time series using Taylor hypothesis^46^ for flows with a characteristic turbulent velocity fluctuation $\le 10 \%$ of its mean convection velocity^47^, as:

13 $$\begin{aligned} L_u = T_u \times U, \end{aligned}$$

where *U* is the convection velocity, and the integral time scale $T_u$ is defined as:

14 $$\begin{aligned} T_u=\int _0^{t_0} \rho _{uu}(\tau ) \,d \tau , \end{aligned}$$

here, $t_0$ is the time lag where the correlation becomes sufficiently low^48,49^, typically set such that $\rho _{uu}(t_0) \le 0.01$.

The approximations for the transition frequency (Eq. 11) indicate that as the scaling constant *c* increases, the transition frequency decreases. This is illustrated by considering the spectral density of a LRD covariance function for various *c* values in Fig. 2b, while keeping all other parameters constant. The transition frequency and integral time/length scale results for short-range dependence (SRD) are provided in the Supplementary Material.

## Procedure to obtain turbulence spectrum via proposed spectrum model

This section details the procedure for obtaining the five required parameters–$\alpha , \beta , \gamma , \sigma ^2$, and *c*–in Equation 1 for the spectrum model. Each step is thoroughly illustrated using atmospheric boundary layer (ABL) and tidal flow velocity measurements as examples. The procedure comprises six main steps:

1. Preprocess the measured or simulated time series using an Empirical Mode Decomposition (EMD)-based sifting method to detrend the data, ensuring the time series is wide-sense stationary (WSS) before calculating the required turbulence quantities.
2. Obtain the variance $\sigma ^2$, integral time scale $T_u$, and integral length scale $L_u$ from the detrended WSS time series.
3. The fractal dimension (*D*) of a stochastic process can also be estimated using various techniques, such as variogram estimators^50^. For high Reynolds number turbulence spectra, typically $D \approx 1.7 \pm 0.3$^40–42^.
4. Conduct a Multifractal Detrended Fluctuation Analysis (MF-DFA) to determine the Hurst component, *H*, and the multifractal spectrum width $\Delta h_q$ of the measured or simulated time series. The monofractal Hurst component *H* will determine the energy-containing scaling in the turbulence spectrum^13,14,21^.
5. Establish the parameter $\gamma$ using the empirical multifractal spectrum width-$\gamma$ relationship depicted in Fig. 1c.
6. Once $\sigma ^2$, $T_u$ ($L_u$), $\gamma$, *D* and *H* are obtained, calculate $\alpha , \beta$, and *c* using Equations 9, 10 and 11.

Detailed methodology explaining these steps are discussed in the Materials and Methods section^51^.

**Fig. 3:**
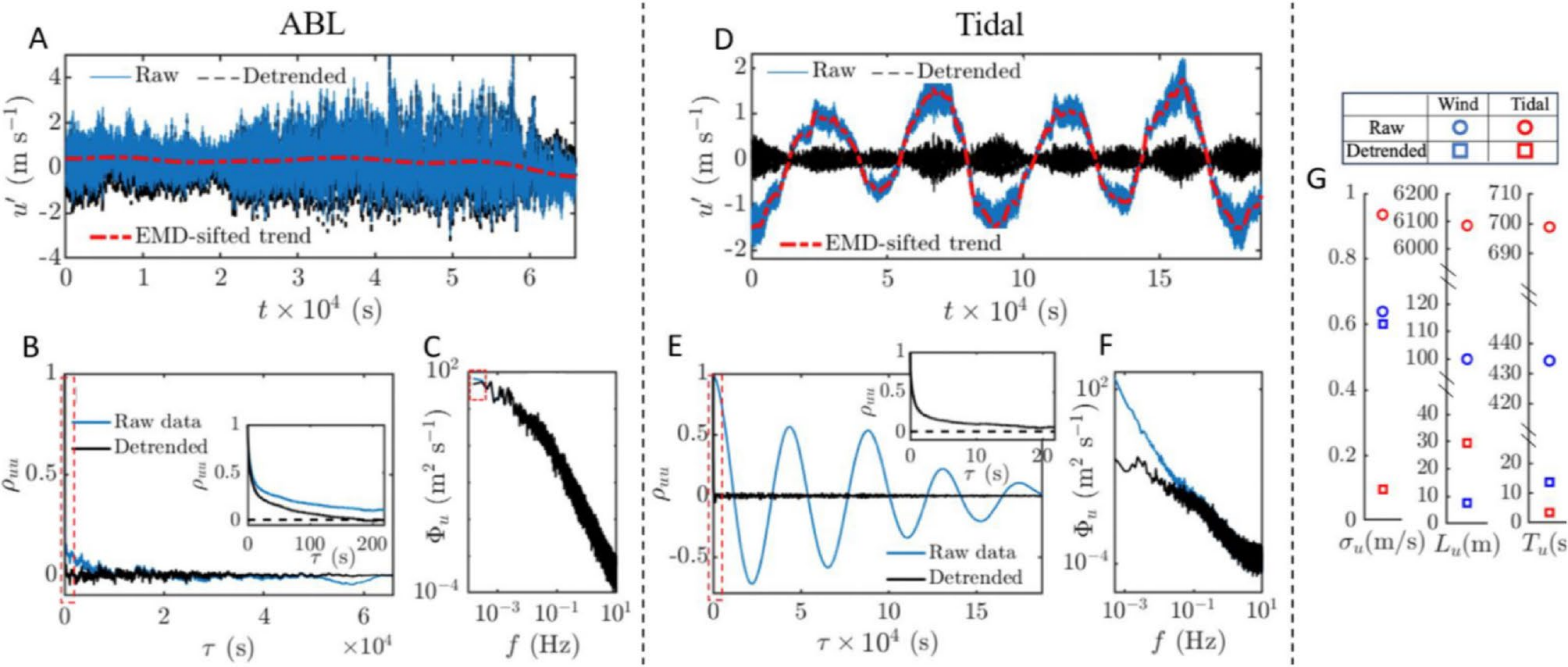
(**a**,**d**) Sample atmospheric boundary layer data and tidal flow data with their background trend obtained by EMD sifting method as introduced in^26^. Comparison between quantities computed using detrended and raw time series; (**b**,**e**) autocorrelation $\rho _{uu}$, (**c**,**f**) streamwise velocity spectrum $\Phi _u$, and (**g**) $\sigma _u, T_u$ and $L_u$ obtained using raw and detrended datasets.

**Fig. 4:**
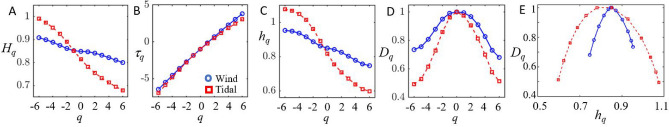
(**a**) Generalized Hurst component, $H_q$, (**b**) $q-$th order mass exponent, $\tau _q$, (**c**) $q-$th order singularity exponent, $h_q$, (**d**) $q-$th order singularity dimension, $D_q$, and (**e**) multifractal spectrum for the wind (blue solid line) and tidal (red dashed line) data.

## Results and discussion

Two distinct sets of experimental turbulence data, from tidal currents and the atmospheric boundary layer (ABL), are used to compare and evaluate the velocity spectrum against traditional turbulence models and the proposed formulation. A set of atmospheric turbulence data was obtained using a CSAT3 sonic anemometer positioned on a meteorological tower at a height of 80 m and sampled over 24 hours at a frequency of 20 Hz. The meteorological tower is located at the University of Minnesota Eolos Wind Energy Research Field Station. For more details on the atmospheric boundary layer data, refer to Chamorro *et al.*^52^. The tidal current data were obtained from the Nodule Point, WA, tidal energy site. This dataset was collected using an Acoustic Doppler Velocimeter (ADV) during the spring tide of 2011, with a sampling frequency of $f_s = 32$ Hz over a period of 4.3 days. The ADV was positioned at the apex of the Tidal Turbulence Tripod, 4.7 m above the seabed, which was 22 m deep at Nodule Point, located on the eastern side of Marrowstone Island. The unprocessed ADV data underwent the phase-space-thresholding (PST) test^53^ to detect spikes and outliers, which were replaced by time-averaged values. For more information on the sampling parameters and Doppler noise, refer to Thomson *et al.*^54^.

### Detrended time series and turbulence quantities

Figure 3a,d illustrates the raw time series alongside the EMD residue and the detrended time series. For the ABL dataset, the residue appears as a moving mean with slight deviations from a constant zero mean, while large-scale motions dominates the tidal flow residue. These results highlight the effectiveness of EMD in eliminating both moving-mean and large-scale trends.

The impact of detrending on turbulence quantities is demonstrated by comparing the streamwise velocity autocorrelation function, $\rho _{uu}$, and their Fourier domain spectrum ($\Phi _u$). The streamwise velocity autocorrelation $\rho _{uu}$ is defined by Equation 15^55^:

15 $$\begin{aligned} \rho _{uu}(\tau )=\overline{u^{\prime }(t) u^{\prime }(t-\tau )} / \sigma _u^2, \end{aligned}$$

where $u' = u - U$ denotes the streamwise velocity fluctuations, $U = \langle u \rangle$ the mean velocity, $\tau$ the time lag, and $\sigma _u^2$ the variance of the signal.

Despite only slight background residue shifts, the ABL dataset demonstrates that proper detrending significantly affects the autocorrelation function $\rho _{uu}$ (Fig. 3b), influencing the estimation of variance $\sigma ^2$, integral length scale $L_u$, and integral time scale $T_u$ (Fig. 3g). For non-detrended time series, $\rho _{uu}$ decays very slowly, not approaching zero until $\tau \approx 3700$ s and 17000 s for ABL and tidal velocity data, respectively (Fig. 3b,e). A closer examination of the highlighted region for $\tau \le 200$ seconds in the inset of Fig. 3b reveals that the raw data $\rho _{uu}$ quickly decays to a plateau value of approximately 0.2 and drops very slowly thereafter, indicating the non-ergodic and non-stationary nature of the original time series^56,57^. A similar effect is observed for the tidal flow data in Fig. 3e, where the large-scale periodic oscillation in $\rho _{uu}$ reflects the large-scale motions of the tidal currents. In contrast, the detrended $\rho _{uu}$ decays quickly to zero as highlighted in the insets of Fig. 3b,e. The estimations of $\sigma _u, T_u$, and $L_u$ using raw and detrended time series are shown in Fig. 3g, revealing a significant overestimation of these quantities by an order when using the raw signal due to imposed mean on a non-stationary signal, leading to erroneous large-scale fluctuations. Conversely, accurate estimations from the detrended data show $L_u$ and $T_u$ values consistent with the inverse of the transition frequency $1/z_T$ from the spectrum and match those reported for ABL and riverine/tidal data under similar conditions and velocity ranges^54,58–61^.

The effect of removing background residue also significantly influences the velocity spectrum at the ,largest scales (i.e., the low frequency component), where high energy level in the raw $\Phi _u$ is observed due to non-stationarity; specifically, $f \le 10^{-3}$ Hz for ABL and $f \le 10^{-1}$ Hz for tidal flows as shown in Fig. 3c,f. Since EMD segregates intrinsic mode functions and residue by scale order, subtracting the residue predominantly affects the velocity spectrum only at the largest scales. This effect is demonstrated by the overlap of the raw and detrended higher frequency $\Phi _u$ components in Fig. 3c,f. The overlap highlights the efficacy of the EMD detrending algorithm, which accurately removes large-scale trends while preserving the integrity of smaller-scale fluctuations. This attribute showcases the advantages of EMD over other regression-based and frequency-based filtering methods for turbulence studies^26^.

**Fig. 5:**
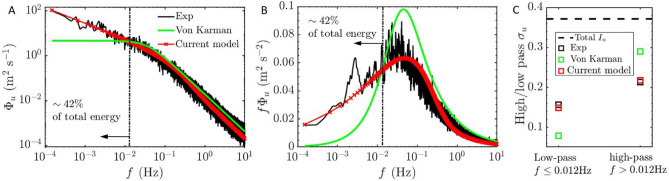
Comparison between experimental measurement, Von Kármán model, and proposed spectral model for atmospheric boundary layer flow; (**a**) streamwise velocity spectrum $\Phi _u$, (**b**) premultiplied spectrum $f\Phi _u$, and (**c**) band-/ and low-pass $\sigma _u$.

**Fig. 6:**
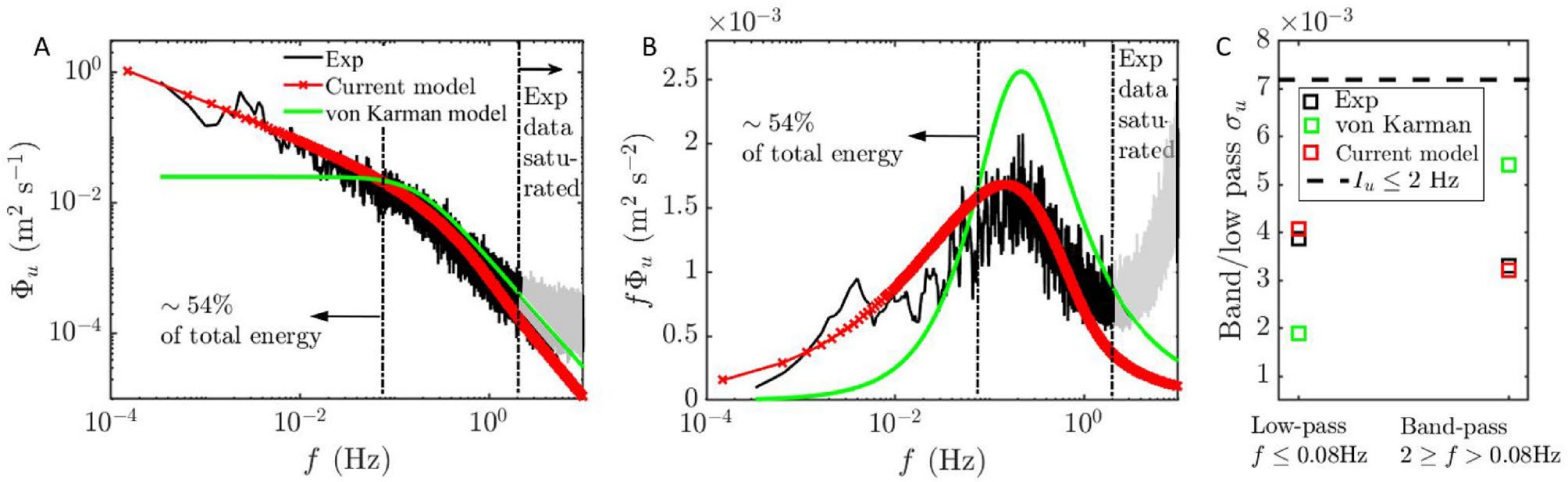
Comparison between experimental measurement, Von Kármán model, and proposed spectral model for tidal flow; (**a**) streamwise velocity spectrum $\Phi _u$, (**b**) premultiplied spectrum $f\Phi _u$, and (**c**) high-/ and low-pass $\sigma _u$.

### DFA and multifractal spectrum results

The distributions of the generalized Hurst exponent ($H_q$), mass exponent ($\tau _q$), singularity exponent ($h_q$), singularity dimension ($D_q$), and the multifractal spectrum for ABL and tidal data are illustrated in Fig. 4. The generalized Hurst component, shown in Fig. 4a, demonstrates a monofractal Hurst component $H > 0.5$ for both datasets, confirming the LRD effect. The non-flat distribution of $H_q$ also suggests significant multifractality for both ABL and tidal flows, corroborated by the multifractal spectrum in Fig. 4e. The multifractal spectrum permits the extraction of the Hurst component $H = H_{q=1}$ and the multifractal spectrum width $\Delta h_q$. Here, a wider $\Delta h_q$ but a lower *H* is observed for tidal flow, indicating stronger multifractality but lower LRD compared to the ABL dataset.

### Model parameters

Using the turbulence statistics and the multifractal spectrum, it is possible to derive all required parameters, construct the covariance function class of Equation 1, and obtain the modeled spectrum.

For the detrended ABL and tidal flow datasets, the derived parameters are as follows: $H = [0.85 \pm 0.03, 0.78 \pm 0.02]$, $\Delta h_q = [0.21 \pm 0.02, 0.48 \pm 0.01]$, $\sigma _u^2 = [3.64 \pm 0.03, 0.82 \pm 0.02] \times 10^{-1}$ m/s, $T_u = [30.05 \pm 0.03, 3.34 \pm 0.01]$ s, and $D = [1.68 \pm 0.04, 1.70 \pm 0.02]$ estimated using both box-counting and variogram methods, which agrees well with those reported in the literature^40–42^. The Hurst components $>0.5$ indicating LRD for both flows, where riverine flows often shows LRD resulting from hydrological processes and climatic variability^15–17^. Similarly, turbulent flows in environmental boundary layers exhibit long-term persistence influenced by large-scale weather systems^18^, external forcing mechanisms^19^, and atmospheric stability conditions^20^.

Following the procedure outlined in Section 2, $\alpha$ and $\beta$ are determined using Equations 9 and 10 with a selected $\gamma = 2$, resulting in $\alpha = [0.32 \pm 0.04, 0.30 \pm 0.02], \beta = [0.15 \pm 0.03, 0.22 \pm 0.02]$, where the uncertainty in parameter estimation are inherent from flow statistics, and Equation 11 leads to $c = [6.64, 0.84]$ for ABL and tidal data respectively. The choice of $\gamma = 2$ is motivated by the high degree of multifractality demonstrated in both datasets (Fig. 4b). For additional results with lower $\gamma$ values, please refer to the Supplementary Material.

### Model performance evaluation

With the derived parameters, the modeled proposed spectra are compared with the experimentally measured spectra and the classical von Kármán spectra in Figs. 5 and  6.

The experimentally measured spectra in Figs. 5a and  6a exhibit negative scaling in the energy-containing range, demonstrating the LRD effect^14,21,62–64^. In contrast, the widely used IEC von Kármán spectral model fails to capture the correct scaling within this range due to its inability to model LRD effects. It shows a flat scaling and underestimates the energy level within the energy-containing range.

It is critical to note that large-scale energy-containing range eddies contribute significantly to the velocity variance–approximately 40% and 55% for ABL and tidal flow datasets, respectively (black dashed-line in Figs. 5a,b and  6a,b). These eddies are crucial for wind and tidal turbine design and operation^65–67^, as studies have shown that background flow structures with scales larger than the wind turbine rotor diameter significantly affect turbine power and wake velocity fluctuations^68–70^.

Moreover, the consequences of inaccurate energy-containing range scaling by the von Kármán spectral model are further illustrated by the compensated spectrum. There is a clear overestimation in the high-frequency bands of the pre-multiplied von Kármán spectra in Figs. 5b and  6b, as the model compensates for the underestimated energy level within the energy-containing range to match the full band variance with the detrended time series.

The enhanced performance of the proposed model relative to the IEC von Kármán spectral model is demonstrated in Figs. 5c and 6c. The omission of long-range dependence (LRD) effects in the von Kármán model leads to an underestimation of energy in the energy-containing range, as quantified by the low-pass-filtered standard deviation $\sigma _u$. Specifically, the von Kármán model captures only 48% and 53% of the experimentally measured low-pass $\sigma _u$ for the ABL and tidal spectra, respectively.

In contrast, the proposed spectral model incorporates LRD effects and offers tunable parameters *D* and *H*, enabling accurate representation of energy levels across all frequency ranges, including precise control of the transition frequency via the scaling parameter *c*. The resulting low-pass $\sigma _u$ deviates from the experimental measurements by only 5% for the ABL and tidal spectra, indicating a substantial improvement in predictive accuracy over the IEC von Kármán model.

## Conclusions

The findings from this study indicate that the proposed turbulence spectrum model provides a significant advancement in modeling field turbulence spectra across diverse environmental conditions. This model, derived from a newly developed class of covariance functions, offers several distinct advantages over traditional turbulence spectrum models, such as the IEC von Kármán isotropic model and the IEC Kaimal model.

The proposed model introduces parameters that explicitly account for long-range dependence and fractal dimension. This differentiation is critical as it allows for a more nuanced representation of turbulence dynamics, particularly in capturing the negative slope of the spectrum in the energy-containing range (that are linked to LRD of the flows^12–14^), a feature often observed in atmospheric and oceanic turbulence but inadequately represented by classical models. The inclusion of the LRD effect and the ability to decouple between Hurst exponent *H* and fractal dimension *D* by the proposed model results in better accuracy in energy level representation across all scales compared to previous turbulence spectrum models. This precision is not merely a theoretical enhancement but has practical implications in improving the fidelity of simulations used in wind and tidal energy research, where accurate energy predictions are crucial for design and operational efficiency. Also, it has been recently shown that, under statistically isotropic conditions, the fractal and Hurst characteristics of the lateral velocity components are identical to those of the streamwise component^71^. This offers a natural extension of the proposed approach to modeling full velocity fields. Another significant benefit of the proposed model is its ease of use. The model is designed with a clear and simple procedural approach that minimizes the need for non-trivial parameter tuning. This user-friendly aspect makes it accessible to practitioners and researchers who may not specialize in the theoretical aspects of turbulence modeling.

It is important to note that the proposed model, due to the monofractal structure of Equation 1, is limited in its ability to capture the multifractal and heavy-tailed characteristics of turbulent velocity fluctuations. Future work will aim to extend the underlying Gaussian random field to a superstatistical framework^72^, enabling improved representation of intermittency and progression toward a multifractal formulation^73^. Since the turbulence spectrum fundamentally governs the distribution of turbulent kinetic energy across scales, it plays a critical role in stochastic, full-field turbulence simulators. In this context, implementing the proposed model in tools such as TurbSim, replacing conventional spectra like the IEC von Kármán and Kaimal models, can yield more accurate simulations of environmental flows that exhibit long-range dependence (LRD). The proposed model improves spectral fidelity across all frequency ranges and significantly reduces band-pass $\sigma _u$ errors compared to classical models currently used in TurbSim^74^, thereby enhancing the accuracy of turbine load predictions and levelized cost of energy (LCOE) assessments^7,9^. These improvements underscore the model’s potential to substantially impact the planning and implementation of wind and tidal energy plants. Its ability to provide more accurate predictions of turbulence behavior will likely lead to enhancements in turbine design, site selection, and overall energy efficiency. The broader adoption of this model could, therefore, contribute significantly to optimizing renewable energy resources, aligning with global sustainability goals.

## Methods

### Empirical mode decomposition (EMD) based sifting method for detrending

Atmospheric boundary layer, tidal, and riverine flows are typically non-stationary. Data detrending is essential to ensure time series data are wide-sense stationary (WSS) before assessing turbulence statistics^26^ such as variance ($\sigma ^2$), turbulence intensity level ($I_u$), and integral time/length scale ($T_u,L_u$), which serve as input parameters for the proposed spectrum model. We adopt a fast and adaptive EMD-based sifting method^75^ to detrend the time series data *x*(*t*), with an overview of the algorithm provided below:

1. Identify the local extrema of the time series. The local minima and maxima are connected using cubic splines to construct the lower and upper envelopes.
2. Average the lower and upper envelopes constructed in the previous step to form a new mean envelope, $m_1(t)$. This is the first intrinsic mode function (IMF) that contains the highest frequency fluctuations within *x*(*t*).
3. Compute the difference $h_1(t)$ between the original signal *x*(*t*) and the first IMF $m_1(t)$:
  16 $$\begin{aligned} h_1(t) = x(t) - m_1(t). \end{aligned}$$
4. Repeat the first two steps using the difference signal $h_1(t)$ as a new signal, and generate the second IMF $m_2(t)$ by computing a new mean from the upper and lower envelopes of $h_1(t)$.
5. The second iteration yields a new difference signal $h_2(t)$:
  17 $$\begin{aligned} h_2(t) = h_1(t) - m_2(t). \end{aligned}$$
6. Store the *n*-th order IMFs, $m_n(t)$, and iterate through the above process until a stopping criterion indicates that no more fluctuating IMFs can be sifted. The last difference signal $h_n(t)$ is termed the residue, which should show no significant variation upon further iteration.
7. Once the residue $h_n(t)$ is obtained, compute the detrended time series $x_{d}(t)$:
  18 $$\begin{aligned} x_{d}(t) = x(t) - h_n(t). \end{aligned}$$

#### Hurst exponent using detrended fluctuation analysis

Multifractal Detrended Fluctuation Analysis (MF-DFA) is performed to obtain the Hurst component that characterizes the degree of Long-Range Dependence (LRD) within a time series. MF-DFA is a robust tool for extracting scale-invariant structures within time series^45,76,77^ and is required here to incorporate the LRD within the proposed spectrum model, where classical spectrum models ignore the LRD effect (i.e., assuming that $H = 0.5$). This assumption used in classical spectrum models will later be proved incorrect and will be addressed by the proposed spectrum model. The fractal analysis of a time series $x_k(t)$ is conducted to determine the Hurst exponent *H* and the multifractal spectrum width $\Delta h_q$. MF-DFA involves the following steps:

1. Generate a random walk-like time series $X_i$ from a noise-like time series $x_k(t)$ by
  19 $$\begin{aligned} X_i = \sum _{k=1}^i \left( x_k - \langle x_k \rangle \right) , \quad i=1, \ldots , N. \end{aligned}$$
2. Divide $X_i$ into $N_s = \text {int}(N/s)$ non-overlapping intervals of equal timescale *s*.
3. Remove the local trend from each of the $N_s$ segments by subtracting the residues obtained via the EMD-based sifting method.
4. Calculate the variance for each segment $v = 1, \ldots , N_s$, where the variance is defined as:
  20 $$\begin{aligned} F^2(s, v) = \frac{1}{s} \sum _{i=1}^s \left\{ X[(v-1)s + i] - x_v(i)\right\} ^2, \end{aligned}$$
  and $x_v(i)$ represents the EMD residue in the *v*-th segment.
5. Compute the *q*-th order fluctuation function by:
  21 $$\begin{aligned} F_q(s) = \left\{ \frac{1}{N_s} \sum _{v=1}^{N_s} \left[ F^2(s, v)\right] ^{q / 2}\right\} ^{1 / q}, \text {for} \; q \ne 0. \end{aligned}$$
6. Estimate the generalized *q*-th order Hurst exponent $H_q(q)$ by the relation:
  22 $$\begin{aligned} F_q(s) \sim s^{H_q(q)}. \end{aligned}$$
  The first order Hurst component is the monofractal Hurst component *H* (i.e., $H = H_{q=1}$).

#### Multifractal spectrum width $\Delta h_q$ from multifractal spectrum

The multifractal spectrum is derived through a two-step transformation process of the *q*-th order Hurst exponent, $H_q$. Initially, $H_q$ is utilized to compute the *q*-th order mass exponent $\tau _q$ using Equation 23. The derivative of $\tau _q$ yields the *q*-th order singularity exponent ($h_q$), and the *q*-th order singularity dimension ($D_q$) is then derived via a first-order Legendre transformation, as delineated in Equations 24 and 25^76,78,79^. Subsequently, the multifractal spectrum is constructed by relating $h_q$ with $D_q$.

23 $$\begin{aligned} \tau _q = qH_q - 1. \end{aligned}$$

24 $$\begin{aligned} h_q = \frac{\Delta \tau _q}{\Delta q}, \end{aligned}$$

25 $$\begin{aligned} D_q = qh_q - \tau _q. \end{aligned}$$

Two key quantities can be extracted from the multifractal spectrum: $h_{q,0}$, at which the maximum $D_q$ occurs, representing the most dominant Hurst exponent, and the width or range of the $D_q$ spectrum, defined as $\Delta h_q = h_{q,\text {max}} - h_{q,\text {min}}$, indicating the degree of multifractality.

## Supplementary Information

Supplementary Information.

## Data Availability

The datasets generated during and/or analyzed during the current study are available from the corresponding author on reasonable request.
