# Supplementary material for: Incorporating long-range dependence and fractal features in turbulence spectra
Source: Sci Rep. 2025 Aug 27;15:31663. doi: 10.1038/s41598-025-16950-1 (PMC12391321; doi:10.1038/s41598-025-16950-1)
Supplement: Supplementary file 1 — Supplementary Information. [file 41598_2025_16950_MOESM1_ESM.pdf]

Supplementary Information

Incorporating long-range dependence and fractal features in turbulence spectra

Shyuan Cheng, Yaswanth Sai Jetti, Vincent S. Neary, Martin Ostoja-Starzewski, Leonardo P. Chamorro

Short range dependence results

Jetti *et al.* [1] showed that $\psi_{\alpha,\beta,\gamma} \in L_1(\mathbb{R}^d)$, when $\gamma\beta > d$. Thus the new class of covariance functions are not integrable when $\gamma\beta < d$. In other words, $\psi_{\alpha,\beta,\gamma}$ is short range dependent (SRD) when $\gamma\beta > d$ and conversely long range dependent (LRD) when $\gamma\beta \leq d$. Note that for LRD (i.e., $\gamma\beta < d$) the spectral density diverges at zero frequency. This is consistent with the fact that a stationary field with positive covariance is LRD if and only if its spectral density diverges at origin [2]. On the other hand, when $\gamma\beta > d$, the spectral density at zero frequency converges to a constant value and the covariance functions are short range dependent (SRD).

For SRD, the high and low-frequency limit of the spectral density $\widehat{\psi}_{\alpha,\beta,\gamma}$ are given by [3]:

$$\widehat{\psi}_{\alpha,\beta,\gamma}(f) \sim \sigma^2 C_1 (fc)^{-d-\gamma\alpha} \quad \text{for } f \to \infty, \tag{1}$$

$$\widehat{\psi}_{\alpha,\beta,\gamma}(f) \sim \sigma^2 K \quad \text{where } 0 \leq K < \infty \quad \text{if } \gamma\beta > d, \quad f \to 0. \tag{2}$$

$$K = \frac{c^d}{\pi^{d/2} 2^{d-1} \Gamma(d/2)} \int_0^\infty x^{d-1} \left(1 - \left(1 + x^{-\gamma}\right)^{-\alpha}\right)^\beta \mathrm{d}x \tag{3}$$

The above expressions for the asymptotic behavior of spectral density allow us to obtain the transition frequency z_T similar to Eq. 12 in the main text for LRD. When the covariance function is SRD, we use Eq. 1 and 2 to obtain the transition frequency as:

$$z_T = \frac{1}{c} \left(\frac{C_1}{K}\right)^{1/(\gamma\alpha + d)}. \tag{4}$$

Next, we obtain an approximation for transition frequency when the covariance function is SRD using a definition of integral time scale in the temporal domain for comparison. When $d = 1$, the integral time scale, T_u, is obtained as follows:

$$T_u = \frac{1}{\sigma^2} \int_0^\infty \psi_{\alpha,\beta,\gamma}(x)\, dx \tag{5}$$

From the definition of the spectral density, we know that,

$$\hat{\psi}_{\alpha,\beta,\gamma}(0) = \frac{1}{\pi}\int_0^\infty \psi_{\alpha,\beta,\gamma}(x)\,dx \tag{6}$$

By making use of the Equation 2 for approximating $\hat{\psi}_{\alpha,\beta,\gamma}(0)$, we get,

$$T_u = \pi K \tag{7}$$

The spectral density $\widehat{\psi}_{\alpha,\beta,\gamma}(f)$ when $d = 1$, $\gamma\beta = 1.5$, $\gamma\alpha = 2/3$, $\gamma = 2/3$, $\sigma^2 = 1$, and $c = 10$ is illustrated in Figure 1a. The transition frequency approximated as z_T using Eq. 4 and $1/T_u$ calculated using Eq. 7 respectively to validate that $z_T \approx 1/T_u$ for the SRD scenario is also indicated. The transition frequency approximations in Eq. 4, and 7 also indicate that tuning the scaling constant c in SRD has identical effects as in the case of LRD. We find that c controls where the transition between the low and high-frequency regimes occurs (i.e., transition frequency decreases as c increases). This is illustrated in Figure 1b by considering the spectral density of an SRD covariance function for various c values while keeping the $\sigma^2 K$ value and all other parameters constant.

Complementary modeling result with a lower γ

This section provides additional modeling results with a lower γ value selection of $\gamma = 1$. Here, we have $H = h_{q,0} = [0.847, 0.782]$, $\Delta h_q = [0.21, 0.48]$, $\sigma_u^2 = [3.6, 0.8] \times 10^{-1}$ m/s, and $T_u = [30.05, 3.34]$ s for the detrended ABL and tidal flow datasets respectively. For $\gamma = 1$, Equation 9 and 10 in the main text leads to $\alpha = [0.68, 0.68]$, $\beta = [0.31, 0.44]$, and main text Equation 11 gives $c = [6.64, 0.84]$ for ABL and tidal data.

The modeled $\gamma = 1$ ABL and tidal flow spectra are shown in figure 2a,d, where the $\gamma = 2$ results and the experimentally measured spectrum are co-plotted to aid comparison. Both modeled spectra (with $\gamma = 1$ and $\gamma = 2$) captured the negative scaling in the energy-containing range and demonstrated superior accuracy across all frequency ranges compared to those modeled by the classical IEC von Kármán spectral model.

The slight difference between the modeled $\gamma = 1$ and $\gamma = 2$ spectra is illustrated by the compensated spectrum in figure 2b,e, where a smoother transition from the energy-containing range scaling to the asymptotic high-frequency scaling is seen for $\gamma = 1$ case as expected. This slower transition leads to a larger deviation from the experimentally measured ABL spectrum evidenced by the band-passed σ_u reported as figure 2c,f; whereas the tidal flow case shows no significant difference in the band-passed σ_u between $\gamma = 1$ and $\gamma = 2$ cases. + It is worth highlighting that the $\approx 10\%$ band-passed σ_u deviation for the $\gamma = 1$ ABL spectrum in the ABL dataset is accounted for a wide frequency band spanning over two decades (i.e., $f \in [0.012,\ 10]$ Hz), and we demonstrate that the proposed spectrum model regardingand γ value selection has significantly outperformed the $\approx 50\%$ band-passed σ_u deviation shown for the modeled von Kármán spectrum.

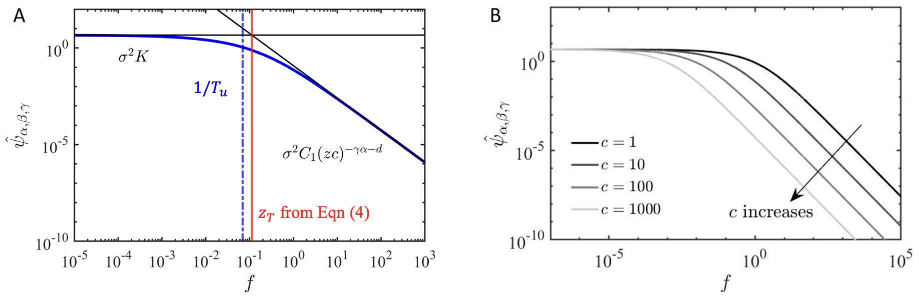

Figure 1: Spectral density $\widehat{\psi}_{\alpha,\beta,\gamma}(f)$ for a short-range dependent (SRD) covariance model. (a) $\gamma\beta = 1.5$, $c = 10$, and $\sigma^2 = 1$, illustrating the transition frequency $z_T \approx 1/T_u$. Black lines indicate the asymptotic low and high-frequency behaviors of $\widehat{\psi}_{\alpha,\beta,\gamma}(f)$. (b) $\gamma\beta = 1.5$ with varying scaling constants c, such that $\sigma^2 K = 4.58$. In all cases, $d = 1$, $\gamma\alpha = 2/3$, and $\gamma = 2/3$.

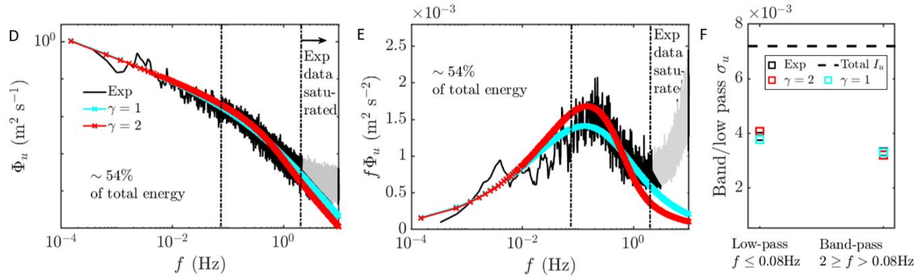

Figure 2: Comparison between experimental measurement, and the proposed spectral model with different γ values for a,b,c) atmospheric boundary layer flow, and d,e,f) tidal flow; a,d) streamwise velocity spectrum Φ_u, b,e) premultiplied spectrum $f\Phi_u$, and c,f) band-/ and low-pass σ_u.

References

[1] Jetti YS, Porcu E, Ostoja-Starzewski M. New decouplers of fractal dimension and Hurst effects. Z für Angew Math Phys. 2023;74(3):123.

[2] Lim S, Teo LP. Gaussian fields and Gaussian sheets with generalized Cauchy covariance structure. Stoch Process their Appl. 2009;119(4):1325-56.

[3] Jetti YS, Shyuan C, Porcu E, Chamorro LP, Ostoja-Starzewski M. A covariance function with fractal, Hurst, and scale-bridging effects for random surfaces and turbulence. Zeitschrift für angewandte Mathematik und Physik. 2025;76(59).
